# The Link between Overweight/Obesity and Noncommunicable Diseases in Ethiopia: Evidences from Nationwide WHO STEPS Survey 2015

**DOI:** 10.1155/2023/2199853

**Published:** 2023-11-16

**Authors:** Bedilu Alamirie Ejigu, Fentanesh Nibret Tiruneh

**Affiliations:** ^1^Department of Statistics, Addis Ababa University, Addis Ababa, Ethiopia; ^2^Department of Applied Human Nutrition, Faculty of Chemical and Food Engineering, Bahir Dar Institute of Technology, Bahir Dar University, Bahir Dar, Ethiopia

## Abstract

**Background:**

Noncommunicable diseases (NCDs) are the leading cause of death worldwide. Each year, 15 million adults die from NCDs; more than 85% of these premature deaths occur in low- and middle-income nations. Evidence indicates that overweight and obesity are the main risk factors for NCDs. Although the literature indicates that the burden of NCDs is increasing in Ethiopia, no research has been conducted to demonstrate a link between overweight/obesity and NCDs. Therefore, the aim of this study is to examine the association between overweight/obesity and the common NCDs while adjusting for other important factors.

**Methods:**

We analysed data from the 2015 Ethiopia WHO STEPS survey, which was conducted in 2015. A total weighted sample of 9,800 participants (15–69 years) was included. The relationship between nutritional status and NCDs was assessed using bivariate and multivariable logistic regression models while adjusting for covariates.

**Results:**

Among the 9,800 participants, 2053 (21% with (95% CI: 19.8–22.1) had hypertension and 1368 (14% with (95% CI: 13.1–15.0) had high cholesterol levels. According to the multivariable logistic regression analysis, being overweight/obese (AOR = 2.0; 95% CI: 1.7–2.3), alcohol consumption, received lifestyle advice, being female, living in urban areas, increased age, having government occupation, and living in SNNP region were positively associated with hypertension. While being underweight (AOR = 0.6; 95% CI: 0.5–0.7), living in the Afar, Somali, and Tigray regions were negatively associated with hypertension. Being overweight/obese (AOR = 1.4; 95% CI: 1.1–1.7), being female, having older age, and living in Somali region were positively associated with a high cholesterol level. Whereas being underweight (AOR = 0.7; 95% CI: 0.6–0.9), received lifestyle advice, reside in rural areas, being farmer, student, and housewife, and living in Gambela region were negatively associated with a high cholesterol level.

**Conclusion:**

This study found a statistically positive association between the common NCDs, namely, overweight/obesity, hypertension, and high cholesterol levels. Our findings imply that there is a need for effective interventions to prevent overweight/obesity by encouraging people to increase physical activity, minimize sedentary behavior, and maintain a healthy dietary pattern in order to reduce the risk of hypertension and high cholesterol levels.

## 1. Introduction

Noncommunicable diseases (NCDs) are the leading cause of death worldwide, accounting for 74% of all deaths. Around 77% of all deaths occur in low- and middle-income countries (LMICs). Each year, 15 million adults die from NCDs globally; more than 85% of these premature deaths occur in LMICs [1, 2]. Studies showed that by 2035, NCDs will be the leading cause of morbidity and mortality in sub-Saharan Africa (SSA) [3, 4].

Likewise, the disease burden and mortality associated with NCDs have increased in Ethiopia over the last few decades [5]. NCD deaths are estimated to be around 42% in the country. Among these, 27% are premature deaths. Disability Adjusted Life Years due to NCDs have increased in the country from 20% in 1990 to 69% in 2015 [6]. If nothing is done to address the problem, Ethiopia will face a staggering burden of early deaths and disability from NCDs by 2040 [7].

Overweight and obesity are the key risk factors for chronic NCDs such as hypertension, hyperlipidemia, type 2 diabetes mellitus, cardiovascular disease, metabolic syndrome, high cholesterol levels, and cancer. High body mass index (BMI) contributes considerably to NCDs including hypertension and all causes of death [8, 9]. Evidence indicates an imbalance between calorie intake and energy expenditure, resulting in adult and adolescent overweight and obesity which is the risk factor of NCDs [10]. Around 4 million deaths and 120 million disability-adjusted life-years were attributed to body weight worldwide [11]. Overweight and obesity affect people of all ages in both developed and LMICs, irrespective of their socioeconomic status [12].

Many LMICs have now entered the nutrition transition stage, which is characterised by increased intake of ultraprocessed foods and large declines in physical activity. This stage is accompanied by rapid rises in the prevalence of overweight-obesity and other nutrition-related NCDs, such as diabetes, hypertension, and high cholesterol levels [13–17].

Overweight and obesity are emerging public health problems in Ethiopia. According to the 2016 Ethiopian Demographic and Health Survey report, the proportion of women who are overweight or obese rose from 3% in 2000 to 8% in 2016. The prevalence of overweight and obesity among adult men increased from 2.5% in 2011 to 3.5% in 2016. However, there is a significant urban-rural disparity in the trend of overweight and obesity. The prevalence in urban areas increased from 7.6% in 2011 to 12.4% in 2016, while rural areas maintained a prevalence of less than 1% [18]. According to recent evidence, the prevalence of overweight and obesity in urban areas was 22.4% and 6.2%, respectively, [19].

Weight loss can improve life expectancy and prevent the onset of NCDs in obese patients [20, 21]. As a result, preventive nutrition, a critical branch of nutrition science, is especially important in the treatment, regression, and prevention of NCDs linked to overweight and obesity [9]. However, there is evidence gap in Ethiopia regarding the association between nutritional status and NCDs such as hypertension and high cholesterol. Previous studies in the country about risk factors for hypertension and cholesterol levels have focused on particular region/district or conducted among subpopulations, limiting their generalizability [22–27]. Most of these studies focused only on sociodemographic and economic factors but did not examine the nutrition-related factors such as nutritional status/BMI. Therefore, this study used data from a nationally representative WHO STEPs survey in Ethiopia to examine the link between nutritional status and the common NCDs while adjusting for other important covariates. We hypothesised that being overweight or obese increased the risk of developing NCDs.

Evidence on the association between nutritional status and NCDS could aid in predicting and tracking the evolution of the NCDs in Ethiopia. In addition, evidence on the national-level risk factor for NCDs can assist policymakers in designing targeted and effective interventions to reduce NCDs morbidity and mortality and improve population health.

## 2. Methods

### 2.1. Study Settings

Ethiopia is a landlocked country located in the Horn of Africa. It shares borders with Eritrea to the north, Djibouti to the northeast, Somalia to the east and northeast, Kenya to the south, South Sudan to the west, and Sudan to the northwest. It is home to around 113.5 million people as of 2022, making it the 13th most populous country in the world, the second most populous in Africa after Nigeria, and the most populated landlocked country on the planet [28].

Even though, Ethiopia has made some progress towards achieving the target for stunting and wasting, but 36.8% and 6.8% of children under 5 years of age are still affected, respectively. It has shown limited progress towards achieving the diet-related noncommunicable disease (NCD) targets. 8.5% women and 2.4% of adult men are living with obesity [29].

### 2.2. Study Design and Sampling Procedures

In this community-based cross-sectional study, we analysed data from the first WHO-STEPs survey in Ethiopia, which was conducted between 14 April and 26 June 2015. We received authorization from WHO STEPs survey after writing a brief proposal outlining regarding the purpose of our study (https://extranet.who.int/ncdsmicrodata/index.php/auth/login/?destination=access_licensed/track/1516). The survey aimed to inform policymakers in order to design evidence-based public health interventions to prevent and control NCD epidemics. A total of 9,800 men and women between the ages of 15 and 69 who had lived at their current address for at least six months were included for interview in the WHO STEPS target population. The survey excluded children under the age of 15 and people older than 69 years old and who had lived at their current address less than six months. The overall response rate was 95.5%. The survey used a single population-proportion formula to determine the sample size. A mix of sampling approaches (stratified, three-stage cluster sampling, simple random sampling, and Kish method) was employed to select the study settings and the study participants. The sampling frame was based on the population and housing census conducted in 2007 (CSA, 2008). Further details on the survey implementation can be found elsewhere [30].

### 2.3. Survey Instrument and Data Collection

The survey used the WHO NCD STEPS instrument version 3.1. The questionnaire was divided into three STEPS for measuring NCD risk factors. STEP I included questions about on socio-demographics and economic as well as behavioural characteristics of sampled population, STEP II included physical measurements to determine percentage of the study population with raised blood pressure, overweight, and obesity, and STEP III included biochemical measurements to measure percentage of the study population with diabetes, high blood glucose, and abnormal cholesterol levels. Data were collected by face-to-face interviews and anthropometric and biochemical measurements.

To meet local needs, the questionnaire was modified and optional questions were added. Additional optional questions, such as Khat chewing, were added to the instrument because they were considered locally relevant. All changes were made in accordance with the STEPS manual [31]. Validated questionnaires were translated into local language, namely, Amharic, Tigrigna, and Oromifa. The questionnaire was then translated back into English. Questions were pretested to ensure that they were clear and understandable to respondents.

### 2.4. Measurements

#### 2.4.1. Outcome Variables

This study had two outcome variables: [1] hypertension and [2] high blood cholesterol. We measured both the outcome variables dichotomously. Hypertension was assessed as if the respondent's average 2^nd^ and 3^rd^ measurement of systolic blood pressure was ≥140 mmHg, or the average diastolic BP was ≥90 mmHg [31]. Raised blood cholesterol was measured as whether respondent blood cholesterol was above 190 mg/dl [32].

#### 2.4.2. Explanatory Variables

The body mass index (BMI), calculated as weight in kilograms divided by height squared in meters, was used to assess the nutritional status in this study. A BMI of less than 18.5 kg/m^2^ was considered undernutrition, a BMI of 18.5–24.9 kg/m^2^ was considered normal weight, and a BMI of 25 kg/m^2^ or higher was considered overweight or obese [31].

Self-reported lifestyle factors included in our analysis were consumption of fruit per week (0, 1-2, and ≥3), serving of fruit one of those days (1-2 and ≥3), consumption of vegetables per week (0, 1-2, ≥3), serving of vegetables on one of those days (1-2 and ≥3), consumption of salt (too much, the right amount and too little), consumption of processed food high in salt (always or often, sometimes and never), most often used oil/fat (lard or suet, vegetable oil others, and none used), frequency of meal consumption outside the home (0–2 per week and ≥3), ever consumed alcohol (yes/no), smoking status (current smoker/never smoker), and khat-chewing habit (yes/no).

We measured lifestyle advise based on six yes/no questions whether the respondents got advise from health professionals to prevent NCDs. Respondents were specifically asked “During the past three years, has a doctor or other health worker advised you to do any of the following?” [1] Quit using tobacco or don't start, [2] reduce salt in your diet, [3] eat at least five servings of fruit and/or vegetables each day, [4] reduce fat in your diet, [5] start or do more physical activity, and [6] maintain a healthy body weight or lose weight. If the respondents answer “yes” to the above the average score, then they were categorized as having got advise; otherwise, we categorized them as no advise. Further, in our analysis, a number of socioeconomic and demographic factors including respondents' sex, age, residence, educational status, and work status were controlled.

### 2.5. Statistical Analysis

All analyses were performed using Stata 17.1 [33]. WHO STEPS survey datasets are often complex in nature for two reasons: (i) the use of stratified multistage cluster sampling to increase sampling accuracy and cost efficiency and (ii) unequal probabilities of selection from target-populations for sampled elements, often as a result of oversampling of key subgroups. Thus, during the estimation of NCD proportion, the data analysis tools employed sampling weights for generating unbiased population estimates [33, 34].

### 2.6. Multilevel Logistic Regression Model

The multilevel logistic regression model is a very popular choice for analysis of dichotomous data. Due to the fact that the probability of having NCDs possibly varies in different clusters, a cluster-level random intercept is introduced in the generalized linear mixed model. We conducted a series of bivariate and multivariate analyses by using a multilevel binary logistic regression model to examine the association between explanatory variables with the outcome variables while controlling for confounding factors. Those explanatory variables that showed significance (*p* < 0.25) in the bivariate analysis were included for multivariate analysis. Let *y*_*ij*_ denote the binary outcome for subject *i* in cluster *j* and assume that *y*_*ij*_ follows a Bernoulli distribution with probability of success, *p*_*ij*_. Then, using the usual logit link function, a binary outcome can be associated with a linear predictor as follows:

(1) $$\begin{array}{c} \text{logit}\left(p_{ij}\right)=\beta_{0}+\beta X_{ij}+u_{j}, \end{array}$$

where *β*_0_ is an intercept, *β* is an unknown parameter for individual level predictors, and *u*_*j*_ are mutually independent Gaussian random effects used to capture within-cluster correlation. In standard multilevel models, *u*_*j*_ is usually assumed to be a normally distributed random intercept with mean 0 and variance *σ*_*u*_^2^. The multilevel approach produces reliable standard errors and parameter estimates when outcomes for individuals within clusters are correlated [35, 36].

The goodness of fit of each model was evaluated by using the Hosmer and Lemeshow test. A higher *p* value indicates that the model fits well. The *p* values for all models were >0.05. We examined multicollinearity problems in the regression models by estimating the variance inflation factor and tolerance. All tolerance values were >0.1, and all variance inflation factor values were <10. Therefore, no multicollinearity problems were observed in the regression models.

### 2.7. Ethical Consideration

The WHO STEPS survey data were collected without any personal identifiers. Informed consent was obtained from the study participants before data collection, and objectives of the study were explained to the participants by the data collectors. The WHO STEPS protocols and procedures were reviewed approved by the Ethiopian Public Health Institution (EPHI) institutional review board (IRB) and National research and Ethics review committee (NRERC).

## 3. Results

Table 1 presents the characteristics of our study participants and prevalence of common hypertension and cholesterol level by respondent's socio-demographic, lifestyle, and behavioural factors. About 5336 (54.4%) respondents were male, and the majority of them 7927 (80.9%) lived in rural areas. Significant proportions of respondents were married, had no formal education, and worked as farmer. Regarding lifestyle and behavioural factors, around 5564 (56.8%) and 4114 (42.0%) respondents reported that not consuming fruit and vegetable per week, respectively. Most of the respondents 5912 (60.3%) used salt frequently. About 411 (4.2%) and 1860 (19.0%) respondents reported smoking cigarettes and had Khat chewing habit, respectively. In terms of nutritional status, approximately 25% of respondents were underweight, while 6.3% were overweight or obese. The vast majority of respondents 9169 (93.6%) reported that they did not receive any lifestyle advice for preventing NCDs from healthcare professionals.

Among 9,800 study participants, 2,053 (21% with 95%:19.8–22.1) and 1,368 (14% with 95% CI: 13.1–15) participants had hypertension and high cholesterol levels, respectively. The magnitude of hypertension and high cholesterol levels were higher among females, urban residents, older age groups, college and above educational level, government/skilled private employee, alcohol drinkers, and those who were overweight/obese (Table 1).

Table 2 depicts the results of both bivariate and multivariable multilevel logistic regression analyses. The bivariate analysis results revealed that, with the exception of fruit and vegetable consumption, all considered explanatory variables were significantly associated with hypertension. Similarly, the majority of explanatory factors were associated with high cholesterol levels with exception of dietary intake of fruits and vegetables, frequency of salt consumption, type of oil, alcohol drinking, cigarette smoking, and life style advice.

Our multivariable analysis revealed that, nutritional status (BMI), sex, place of residence, age, educational status; occupation, region, alcohol consumption, and lifestyle advice were significantly associated with hypertension (Table 2).

Nutritional status was positively associated with hypertension. The likelihood of developing hypertension was 2.0 times higher in overweight/obese respondents than in normal weight respondents (AOR = 2.0; 95% CI: 1.7–2.3). On the other hand, being underweight was negatively associated with chance of developing hypertension. The likelihood of developing hypertension was 40% (AOR = 0.6; 95% CI: 0.5–0.7) lower among underweight respondents than in normal-weight respondents. Alcohol consumption was significantly associated with hypertension. The likelihood of developing hypertension was 20% (AOR = 0.8; 95% CI: 0.7–0.9) lower in nondrinkers than in drinkers of alcohol. Respondents who received advice from healthcare professionals regarding NCDs were 1.4 (AOR = 1.4; 95% CI: 1.1–1.7) times more likely to have hypertension than their counterparts.

In terms of socio-demographic factors, being female was positively associated with hypertension and high cholesterol levels. Females were 1.2 times more likely to develop hypertension than males (AOR = 1.2; 95% CI: 1.01–1.32). Living in rural areas was linked to an increased risk of developing hypertension. Rural residents had 0.8 times lower likelihood of hypertension than their urban counterparts (AOR = 0.8; 95% CI: 0.7–0.9). Older age groups were positively linked to hypertension than younger age groups. The likelihood of developing hypertension was 3.6 (AOR = 3.6; 95% CI: 2.9–4.4) times higher for respondents aged 50 and over than for those aged 15 to 24. When compared to respondents with no formal education, those with some educational level had a 0.8 (AOR = 0.8; 95% CI: 0.7–0.9) times lower risk of developing hypertension. Being a farmer, trader, or student was negatively lined with hypertension by 0.7 (AOR = 0.7; 95% CI: 0.6–0.9, AOR = 0.7; 95% CI: 0.5–0.9, and AOR = 0.7; 95% CI: 0.4–0.9) times compared to working for the government or a skilled private employer. Afar, Somali, and Tigray region residents had 0.5, 0.6, and 0.4 (AOR = 0.5; 95% CI: 0.3–0.8; AOR = 0.6; 95% CI: 0.4–0.9; and AOR = 0.4; 95% CI: 0.3–0.6) times lower chance of hypertension than Addis Ababa residents. However, respondents who live in the S.N.N.P region were 1.7 (AOR = 1.7; 95% CI: 1.2–2.3) times more likely to develop hypertension than Addis Ababa residents.

Regarding the outcome of cholesterol levels, nutritional status (BMI), lifestyle advice, sex, residence, age, occupation, and region were found statistically significant factors. Being overweight/obese was positively associated with high cholesterol levels. Overweight/obese respondents had 1.4 times higher chance of having high cholesterol levels than respondents of normal weight (AOR = 1.4; 95% CI: 1.1–1.7). In contrast, being underweight was negatively associated with high cholesterol levels. Respondents who were underweight had a 0.7 (AOR = 0.7; 95% CI: 0.6–0.9) times lower risk of developing high cholesterol levels than those who were of normal weight. Health professional lifestyle recommendations reduced respondents' risk of high cholesterol by 0.8 (AOR = 0.8; 95% CI: 0.6–0.9) times. In comparison to males, females were 1.4 times (AOR = 1.4; 95% CI: 1.2–1.7) more likely to have high cholesterol. Rural residents had a 0.6 (AOR = 0.6; 95% CI: 0.4–0.7) times lower likelihood of having high cholesterol than urban residents. The likelihood of developing higher cholesterol levels was 1.3 and 1.5 times higher for respondents in the age groups of 36–49 and 50 and above, respectively (AOR = 1.3; 95% CI: 11.02–1.6 and AOR = 1.5; 95% CI: 1.2–1.9), compared to respondents in the 15–24 age group. Farmers, students, and house maker/house wives had lower likelihood of having high cholesterol levels than government/skilled private employees by 0.7, 0.6, and 0.7 (AOR = 0.7; 95% CI: 0.6–0.9; AOR = 0.6; 95% CI: 0.5–0.9; and AOR = 0.7; 95% CI: 0.5–0.9) times. Respondents who resided in the Gambela region had 0.4 (AOR = 0.4; 95% CI: 0.2–0.8) times less chance of having a high cholesterol level than respondents who resided in Addis Ababa. In contrast, respondents living in the Somali region were 3.4 times more likely than Addis Ababa residents to develop high cholesterol levels (AOR = 3.4; 95% CI: 2.1–5.6). Form the multilevel modelling cluster-level variance (Table 2), the variance partition coefficient (VPC) is (0.6/0.6+3.29)=0.154 and (0.3/0.3+3.29)=0.083 for hypertension and cholesterol levels, respectively. This indicates that 15.4% and 8.3% of the variance in hypertension and high cholesterol levels can be attributed to differences between clusters, respectively.

## 4. Discussion

Our study aimed to examine associations of overweight/obesity and common NCDs (hypertension and high cholesterol levels) in Ethiopia. To our knowledge, this is the first study to assess the link between overweight/obesity and NCDs in Ethiopia. This study revealed that the magnitude of hypertension and high cholesterol levels was 21% (with 95% CI: 19.8, 22.1) and 14% (with 95% CI: 13.1, 15.0), respectively, with higher prevalence in the urban population compared to the rural population, and among females as opposed to males and increased with older age. Our findings concur with those of the previous systematic review and meta-analysis studies carried out in Ethiopia [37, 38]. Contrary to our results, some single studies carried out in Ethiopia demonstrate a higher prevalence of hypertension and cholesterol levels [39–43]. The reason for the discrepancy may be due to metrological issues; while previous studies focused only on urban areas with an older population, our study took residence into account as well as a broad age range.

In line with previous findings and our hypothesis [44–51], our study showed that being overweight/obesity was significantly associated with common NCDs. Being overweight/obese was positively associated with hypertension and high cholesterol levels. Studies [52–54] suggested that BMI is not just a marker of factors associated with hypertension but is causally related. This relationship has the potential to lead to a number of implications, one of which is that as BMI raises, elevated blood pressure and cholesterol, significant risk factors for cardiovascular disease, become an even more important health issue. On the other hand, our study found that being underweight was negatively associated with both hypertension and high cholesterol levels. Although being underweight carries a high risk of morbidity and mortality, it may be a protective factor for hypertension and high cholesterol levels. Based on our findings, we recommend that NCD prevention interventions in Ethiopia should emphasize promoting secure nutritional practices and physical activity to maintain a healthy body mass index, as overweight/obesity is an emerging issue in developing nations, including Ethiopia.

Unsurprisingly and in line with prior study results [55–57], our study showed that respondents who did not drink alcohol were less likely to develop hypertension than their counterparts. A randomized control trial study also reported that compared to never-drinkers, current and previous alcohol use had a causal effect on hypertension [58]. In light of its effects on population health, alcohol consumption needs more stringent regulation [57].

Unexpectedly, this study discovered that participants who received lifestyle recommendations from healthcare professionals had higher chance of having hypertension than participants who did not receive. The possible explanation for this result may be respondents with hypertension sought advice and receiving it from medical professionals. When giving advice about NCDs, medical professionals may also prioritize patients who have them. Additionally, our study revealed that receiving lifestyle medical advice for preventing NCDs from health professionals protected against having a high cholesterol level. This finding suggests that in order to create effective dietary and physical activity intervention strategies, it may be necessary to evaluate patient-provider communication regarding NCD prevention and management. A study conducted in the United States also recommended that health professionals should be encouraged to suggest lifestyle modifications for adults with high cholesterol in clinical practice given the better lipid profile and lower the CVD risk of adherents [44].

Unlike the findings of other studies [45–50], our study and some previous studies conducted in Ethiopia [51, 59] reported that females were more likely to develop hypertension and a high cholesterol level than males. The possible explanations for this finding include biological and hormonal differences. Routine housework and cultural factors, as is the case with most Ethiopian females, spend more time indoors than males do, make them more likely to be physically inactive, and accumulate fat tissue, which in turn raises their blood pressure and cholesterol levels [56, 60–62]. This study discovered that rural residents had lower odds of having hypertension and a high cholesterol level than their urban counterparts. This might be because urban and rural residents have different levels of physical activity, different lifestyles, and different dietary habits.

Likewise in prior studies [26, 39, 56], our study found that advanced age increases the likelihood of having hypertension and high cholesterol levels. This may be due to the fact that as people age, their vascular systems become less elastic, resulting in stiffer, less flexible blood vessels that raise blood pressure [62]. Furthermore, compared to younger age groups, older age groups engage in less physical activity. Organ systems may malfunction as we age and become less efficient. These issues, along with lifestyle choices, may contribute to an increased blood pressure and cholesterol levels in older adults.

In the present study, participants who had at least some form of education were exposed less odds to develop hypertension than those who had no formal education. Education may increase a person's likelihood of knowing about hypertension and leading a healthier lifestyle. On the other hand, people with no education might not prioritize their health care needs. Subjects with no education levels may exhibit unhealthier lifestyle behaviors, such as unbalanced diet or lack of exercise, which are linked to developing hypertension [63]. Adults with no or lower educational attainment consumed calorically dense food more frequently than those with higher educational attainment [64]. Compared to government/skilled private employees, farmers, traders, and students had lower chance of hypertension. The likelihood of having high cholesterol was also lower among farmers, students, and housemakers/housewives than for government or skilled private employees. Literature shows that different levels of physical activity and lifestyles among occupations could be some of the potential reasons [65]. Our study suggests that daily activities and type of occupation can have a significant impact on health conditions. So, a health assessment that takes into account occupational factors is crucial to lowering the risk of disease including hypertension and cholesterol levels [66].

Our study found that respondents who reside in Afar, Somali, and Tigray regions were less likely to have hypertension than those who reside in Addis Ababa. In contrast, respondents who live in S.N.N.P region were more likely to develop hypertension than those who lived in Addis Ababa. On the other hand, people who lived in Gambela and Somali regions were less and more likely to have high cholesterol levels than those who live in Addis Ababa, respectively. The possible explanation for these results may be people who live in different regions may have different lifestyles and socioeconomic and socio-cultural structures. For example, people who lived in Afar and Somali regions are mostly living a pastoralist lifestyle. These regions are hot and dry climatic conditions that force pastoralist communities to move around constantly in search of grazing land and water; this leads to high physical activity and less resting time and hence reduces their odds to having hypertension than people living in a relatively modern city, Addis Ababa. Because the Gambela region is a relatively rich fruit production area, people who live there may consume more fruits and organic foods, which may minimize their chances of having high cholesterol levels compared to Addis Ababa residents. On the other hand, people who reside in Somali region, since they are nomadic, mostly consume animal product foods which lead to an increase in their cholesterol levels. The study conducted in Brazil also reported that a high intake of animal products in the diet is more closely associated with cardiovascular risk factors [67]. However, further study may clearly explain such variations among geographical regions in Ethiopia.

This study has some limitations. First, since a cross-sectional study design was used, we were unable to establish causal connections between the explanatory and outcome variables. Second, because this study is based on the analysis of secondary records, there may be some confounding variables that we are unable to fully account for. Despite these limitations, this study included a large sample size, so our results might be generalizable to Ethiopian people. We also took into account a number of vital behavioural and lifestyle factors, as well as socio-demographic and economic aspects related to NCDs.

## 5. Conclusion

Our study demonstrated the presence of significant association between overweight/obesity and common NCDs, namely, hypertension and high cholesterol levels. Our study suggested that there is a need for effective interventions to prevent overweight/obesity by encouraging people to increase physical activity, reduce television time, screen time and other sedentary behavior, and maintain healthy dietary pattern in order to reduce the risk of hypertension and high cholesterol levels.

## Figures and Tables

**Table 1:** Sampled characteristics and prevalence of selected NCDs by respondent's socio-demographic, lifestyle, and behavioral characteristics (*n* = 9,800).

Characteristics of respondents	Frequency (%)	Blood pressure		Cholesterol level	
		Normal %	Hypertension %	Normal %	High %
Lifestyle and behavioral factors					
Consumption of fruit per week					
0	5564 (56.8)	80.0	20.0	85.5	14.5
1-2	3107 (31.7)	79.4	20.6	87.0	13.0
≥3	1122 (11.5)	73.4	26.6	86.3	13.7
No. of servings of fruit per day (*n* = 4,237)					
1-2	3235 (76.3)	78.2	21.8	86.7	13.3
≥3	1002 (23.7)	76.7	23.3	87.2	12.8
Consumption of vegetable per week					
0	4114 (42.0)	82.0	18.0	85.6	14.4
1-2	3513 (35.9)	78.5	21.5	86.3	13.7
≥3	2166 (22.1)	74.4	25.6	86.7	13.3
No. of servings of vegetable/day (5,538)					
1-2	4212 (76.1)	76.9	23.1	86.2	13.8
≥3	1326 (23.9)	77.1	22.9	87.5	12.5
Frequency of salt consumption					
Always/often	5912 (60.3)	81.1	18.9	86.1	13.9
Sometimes	1809 (18.5)	73.4	26.6	87.2	12.8
Never	2079 (21.2)	78.1	21.9	84.8	15.2
Processed food consumption					
Always/often	868 (8.9)	80.9	19.1	90.1	9.9
Sometimes	3925 (40.0)	77.2	22.8	87.0	13.0
Never	5007 (51.1)	80.2	19.8	84.6	15.4
Most often used oil/fat					
Vegetable oil	6381 (65.1)	81.1	18.9	86.0	14.0
Lard/butter/margarine/	776 (7.9)	76.8	23.2	89.3	10.7
Other/none in particular	2186 (23.3)	75.9	24.1	85.2	14.8
None used	457 (4.7)	69.5	30.5	84.5	15.5
Consumption of meal outside the home per week					
0–2	9071 (92.6)	79.1	20.9	85.7	14.3
≥3	721 (7.4)	78.8	21.2	90.6	9.4
Current smoker					
Yes	411 (4.2)	77.7	22.3	86.8	13.2
No	9389 (95.2)	79.1	20.9	86.0	14.0
Alcohol consumption					
Yes	4831 (49.8)	77.8	22.2	84.8	15.2
No	4964 (50.7)	80.3	19.7	87.3	12.7
Body mass index					
Underweight	2352 (25.3)	87.2	12.8	89.7	10.3
Normal	6350 (68.4)	78.7	21.3	87.6	12.4
Overweight/obese	582 (6.3)	57.6	42.4	79.1	20.9
Ever chewed khat					
Yes	1860 (19.0)	80.1	19.9	87.6	12.4
No	7930 (81.0)	78.9	21.1	85.7	14.3
Lifestyle advice					
No	9169 (93.6)	79.5	20.5	86.1	13.9
Yes	631 (6.4)	72.9	27.1	85.2	14.8
Socio-demographic and economic factors					
Sex					
Male	5336 (54.4)	79.4	20.6	87.9	12.1
Female	4464 (45.6)	78.6	21.4	83.8	16.2
Residence					
Urban	1873 (19.1)	74.4	25.6	79.8	20.2
Rural	7927 (80.9)	80.1	19.9	87.5	12.5
Age					
15–24	3479 (35.5)	85.5	14.5	87.2	12.8
25−35	3438 (35.1)	79.2	20.8	86.6	13.4
36–49	1637 (16.7)	75.0	25.0	84.0	16.0
≥50	1246 (12.7)	65.7	34.3	83.9	16.1
Marital status					
Single	2784 (28.4)	84.7	15.3	87.2	12.8
Married	6290 (64.2)	77.8	22.2	86.4	13.6
Divorced	726 (7.4)	68.2	31.8	78.9	21.1
Educational level					
No education	4036 (41.2)	77.1	22.9	85.8	14.2
Less than primary	3572 (36.4)	81.8	18.2	86.0	14.0
Primary school	1076 (11.0)	80.2	19.8	87.2	12.8
Secondary school	687 (7.0)	78.5	21.5	87.7	13.3
College and above	429 (4.4)	73.1	26.9	85.1	14.9
Occupation					
Government/skilled private employee	952 (9.7)	68.6	31.4	82.3	17.7
Farmer	4629 (47.2)	79.2	20.8	86.7	13.3
Trader	491 (5.0)	81.1	18.9	87.2	12.8
Student	1648 (16.2)	86.5	13.5	88.3	11.7
Homemaker/house wife	1738 (17.2)	77.1	22.9	83.8	16.2
Unemployed	342 (3.5)		24.0	86.9	13.1
Region					
Addis Ababa	270 (2.8)	72.0	28.0	76.7	23.3
Afar	95 (1.0)	88.6	11.4	83.1	16.9
Amhara	2886 (29.4)	79.6	20.4	83.7	16.3
Benishangul Gumz	97 (1.0)	81.9	18.1	82.2	17.8
Dire Dawa	41 (0.4)	83.6	16.4	59.1	40.9
Gambela	52 (0.5)	80.9	19.1	93.0	7.0
Harari	23 (0.2)	79.3	20.7	76.3	23.7
Oromiya	3490 (35.6)	82.6	17.4	87.0	13.0
S.N.N.P	2064 (21.1)	69.6	30.4	89.4	10.6
Somali	92 (0.9)	80.4	19.6	65.9	34.1
Tigray	690 (0.7)	87.5	12.5	89.7	10.3
Outcome variables					
Raised blood pleasure/hypertension	2053 (21) 95% CI (19.8–22.1)				
Raised cholesterol	1368 (14) 95% CI (13.1–15.0)				

**Table 2:** Multilevel bivariate and multivariable logistic regression analysis of blood pressure and cholesterol levels.

Characteristics of participants	Blood pressure		Cholesterol level	
	COR (95% CI)	AOR (95% CI)	COR (95% CI)	AOR (95% CI)
Lifestyle and behavioral factors				
BMI (ref = normal)				
Underweight	0.5 (0.3–0.8)^*∗∗*^	**0.6 (0.5–0.7)** ^*∗*^	0.3 (0.1–0.6)^*∗∗*^	**0.7 (0.6–0.9)** ^*∗*^
Overweight/obesity	3.8 (3.1–4.6)^*∗∗*^	**2.0 (1.7–2.3)** ^*∗*^	2.2 (1.8–2.8)^*∗∗*^	**1.4 (1.1–1.7)** ^*∗*^
Consumption of fruit per week (ref = 0)				
1-2	0.9 (0.8–1.10)		0.9 (0.8–1.1)	
≥3	1.01 (0.8–1.2)		1.1 (0.8–1.3)	
Consumption of fruit per day (ref = 1-2)				
≥3	1.01 (0.8–1.2)		0.8 (0.6–1.2)	
Consumption of vegetables per week (ref = 0)				
1-2	1.03 (0.9–1.2)	1.01 (0.9–1.2)	1.01 (0.8–1.2)	
≥3	1.2 (1.1–1.4)^*∗∗*^	0.9 (0.8–1.2)	1.1 (0.9–1.3)	
Consumption of vegetables per day (ref = 1-2)				
≥3	1.02 (0.9–1.2)		0.9 (0.8–1.1)	
Frequency of salt consumption (ref = always)				
Sometimes	1.2 (1.1–1.4)^*∗∗*^	0.9 (0.7–1.04)	1.01 (0.8–1.2)	
Never	1.1 (0.9–1.3)^*∗*^	1.2 (0.9–1.4)	1.04 (0.9–1.2)	
Consumption of processed food high in salt (ref = always/often)				
Sometimes	1.2 (0.98–1.4)^*∗*^	1.1 (0.9–1.4)	1.1 (0.9–1.4)	1.1 (0.8–3.1)
Never	1.1 (0.9–1.3)	1.0 (0.8–1.3)	1.2 (0.9–1.5)^*∗*^	1.2 (0.9–5.6)
Most often used oil type (ref = vegetable oil)				
Lard/butter/margarine	1.3 (1.1–1.6)^*∗∗*^	1.1 (0.9–1.4)	1.001 (0.8–1.3)	1.0 (0.7–1.3)
Other/none in particular	1.2 (1.03–1.4)^*∗∗*^	1.0 (0.8–1.2)	1.1 (0.9–1.3)	0.9 (0.8–1.2)
None use	1.3 (1.01–1.8)^*∗∗*^	1.1 (0.8–1.5)	1.1 (0.8–1.6)	1.0 (0.7–1.5)
Consumption of meal outside the home (ref = 0–2 per week)				
≥3 per week	0.9 (0.8–1.3)^*∗*^	0.9 (0.8–1.2)	0.8 (0.6–0.9)^*∗∗*^	0.9 (0.7–1.2)
Alcohol consumption (ref = yes)				
No	0.8 (0.7–0.9)^*∗∗*^	**0.8 (0.7–0.9)** ^*∗*^	1.1 (0.9–1.2)	
Cigarette smoking (ref = yes)				
No	0.9 (0.7–1.2)^*∗*^	0.8 (0.7–1.1)	1.03 (0.8–1.3)	
Khat-chewing habit (ref = yes)				
No	1.5 (1.2–2.1)^*∗∗*^	1.1 (0.9–1.2)	1.1 (0.9–1.3)^*∗*^	1.0 (0.8–1.2)
Lifestyle advice (ref = no)				
Yes	1.6 (1.4–1.9)^*∗∗*^	**1.4 (1.1–1.7)** ^*∗*^	0.9 (0.7–1.8)	**0.8 (0.6–0.9)** ^*∗*^
Socio-demographic and economic factors				
Sex (ref = male)				
Female	1.1 (0.9–1.2)^*∗*^	**1.2 (1.01–1.32)** ^*∗*^	1.5 (1.3–1.7)^*∗∗*^	**1.4 (1.2–1.7)** ^*∗*^
Residence (ref = urban)				
Rural	0.6 (0.5–0.7)^*∗∗*^	**0.8 (0.7–0.9)** ^*∗*^	0.5 (0.4–0.6)^*∗∗*^	**0.6 (0.4–0.7)** ^*∗*^
Age (ref = 15–24)				
25–35	1.5 (1.3–1.7)^*∗∗*^	**1.4 (1.1–1.6)** ^*∗*^	1.1 (0.9–1.3)^*∗*^	1.0 (0.8–1.2)
36–49	2.3 (2.0–2.7)^*∗∗*^	**2.1 (1.6–2.4)** ^*∗*^	1.2 (1.1–1.5)^*∗∗*^	**1.3 (1.02–1.6)** ^*∗*^
≥50	4.00 (3.4–4.7)^*∗∗*^	**3.6 (2.9–4.4)** ^*∗*^	1.4 (1.2–1.8)^*∗∗*^	**1.5 (1.2–1.9)** ^*∗*^
Educational status (ref = no formal education)				
Less than primary	0.7 (0.6–0.8)^*∗∗*^	**0.8 (0.7–0.9)** ^*∗*^	0.9 (0.8–1.1)^*∗*^	1.1 (0.9–1.3)
Primary	0.7 (0.5–0.8)^*∗∗*^	0.9 (0.8–1.2)	0.8 (0.7–1.1)^*∗*^	1.1 (0.8–1.4)
Secondary	0.8 (0.7–1.01)^*∗*^	1.04 (0.8–1.3)	0.9 (0.8–1.2)	1.1 (0.8–1.5)
College and above	0.9 (0.8–1.2)	0.9 (0.7–1.3)	1.1 (0.8–1.4)	1.1 (0.8–1.5)
Occupation (ref = government/skilled private employee)				
Farmer	0.7 (0.6–0.8)^*∗∗*^	**0.7 (0.6–0.9)** ^*∗*^	0.7 (0.5–0.8)^*∗∗*^	**0.7 (0.6–0.9)** ^*∗*^
Trader	0.7 (0.5–0.9)^*∗∗*^	**0.7 (0.5–0.9)** ^*∗*^	0.7 (0.6–0.9)^*∗∗*^	0.7 (0.5–1.0)
Student	0.4 (0.3–0.5)^*∗∗*^	**0.7 (0.4–0.9)** ^*∗*^	0.5 (0.4–0.7)^*∗∗*^	**0.6 (0.5–0.9)** ^*∗*^
Homemaker/housewife	0.8 (0.7–1.01)^*∗*^	0.8 (0.6–1.01)	0.9 (0.8–1.1)	**0.7 (0.5–0.9)** ^*∗*^
Unemployed	0.9 (0.7–1.2)	0.9 (0.7–1.05)	0.9 (0.7–1.3)	0.8 (0.6–1.1)
Region (ref = Addis Ababa)				
Afar	0.3 (0.2–0.4)^*∗∗*^	**0.5 (0.3–0.8)** ^*∗*^	0.8 (0.5–1.3)	1.4 (0.8–2.4)
Amhara	0.6 (0.5–0.8)^*∗∗*^	1.05 (0.8–1.4)	0.6 (0.4–0.9)^*∗∗*^	1.1 (0.7–1.7)
Benishangul Gumz	0.4 (0.3–0.7)^*∗∗*^	0.8 (0.5–1.3)	0.7 (0.4–1.2)^*∗*^	1.3 (0.7–2.3)
Dire Dawa	0.3 (0.2–0.5)^*∗∗*^	0.6 (0.4–1.1)	0.8 (0.4–1.4)	1.2 (0.6–2.4)
Gambela	0.4 (0.3–0.7)^*∗∗*^	0.8 (0.5–1.4)	0.2 (0.1–0.5)^*∗∗*^	**0.4 (0.2–0.8)** ^*∗*^
Harari	0.5 (0.3–0.8)^*∗∗*^	1.0 (0.5–1.7)	0.8 (0.4–1.6)	1.5 (0.7–3.1)
Oromiya	0.5 (0.4–0.7)^*∗∗*^	1.04 (0.7–1.4)	0.5 (0.3–0.6)^*∗∗*^	0.8 (0.6–1.3)
S.N.N.P	0.9 (0.7–1.1)	**1.7 (1.2–2.3)** ^*∗*^	0.4 (0.3–0.6)^*∗∗*^	0.7 (0.4–1.1)
Somali	0.4 (0.3–0.6)^*∗∗*^	**0.6 (0.4−0.09)** ^*∗*^	1.9 (1.2–2.9)^*∗∗*^	**3.4 (2.1–5.6)** ^*∗*^
Tigray	0.3 (0.2–0.4)^*∗∗*^	**0.4 (0.3–0.6)** ^*∗*^	0.4 (0.3–0.6)^*∗∗*^	0.6 (0.4–1.04)
Random effects				
Var (EA)	—	0.3 (0.2–0.4)	—	0.6 (0.4, 0.7)

*Note*. ^*∗∗*^: *P* < 0.05; ^*∗*^: *P* < 0.25 (for bivariate analysis); ^*∗*^: with boldface: *P* < 0.05 for multivariable analysis.

## Data Availability

The datasets generated and/or analysed during the current study are available in the WHO NCD Microdata repository at https://extranet.who.int/ncdsmicrodata/index.php/catalog/794.

## Abbreviations

AOR: Adjusted odds ratio
BMI: Body mass index
CI: Confidence interval
COR: Crude odds ratio
CVD: Cardiovascular disease
EPHI: Ethiopian Public Health Institution
LMICS: Low- and middle-income countries
NCDs: Noncommunicable diseases
S.N.N.P: Southern Nations, Nationalities and Peoples
SSA: Sub-Saharan Africa
WHO: World Health Organization.
